# Relative influence of peak strain delay and peak strain amplitude of non-scarred myocardium in response to cardiac resynchronization therapy: insights from segmental 4D strain analysis

**DOI:** 10.1186/1532-429X-18-S1-O78

**Published:** 2016-01-27

**Authors:** Alessandro Satriano, Aidan K Cornhill, Yoko Mikami, Nowell M Fine, Bobak Heydari, Naeem Merchant, Carmen P Lydell, Andrew G Howarth, Raymond Yee, Teresa A Whitman, James A White

**Affiliations:** 1 grid.22072.350000000419367697Division of Cardiology, School of Medicine, University of Calgary, Calgary, AB Canada; 2 grid.39381.300000000419368884Ivey Business School at Western University, London, ON Canada; 3 Stephenson Cardiac Imaging Centre, Calgary, AB Canada; 4 grid.412745.10000000091321600London Health Science Centre, London, ON Canada; 5 grid.419673.eMedtronic, Inc, Minneapolis, MN USA; 6 grid.22072.350000000419367697Department of Diagnostic Imaging, University of Calgary, Calgary, AB Canada

## Background

The burden and location of replacement fibrosis by Late Gadolinium Enhancement (LGE) MRI is a recognized predictor of non-response in patients undergoing cardiac resynchronization therapy (CRT). However, the capacity of the non-scarred tissue to respond favorably is felt to be dependent upon incremental factors. In this study we explored the utility of 4D strain analysis of non-scarred myocardial tissue to predict response to CRT.

## Methods

43 patients accepted for CRT by standard clinical criteria were enrolled. Pre-procedural imaging was performed at 3T using a standardized CMR protocol inclusive of routine, multi-planar cine imaging and matched LGE imaging. 4D Strain analysis, using a deformation field approach was performed using in-house software (GIUSEPPE) to obtain segmental estimates of Peak and Time to Peak strain. Strains were calculated transmurally and on the endocardial and epicardial surfaces, where applicable, to describe Radial, Circumferential, Longitudinal and Minimum Principal strains. Spatially matched segmental estimates of %LGE were obtained using cvi42 (Circle Cardiovascular, Calgary). Strain measures were analyzed globally and then segmentally with exclusion of any segment with significant fibrosis (≥5%LGE). All patients were followed for 6 months to determine CRT response, defined as a reduction in LVESV of ≥15% by serial echocardiography.

## Results

Mean age was 69.9+/-9.2 with mean LV EF of 25.8+/-6.0%. A total of 28 (65%) patients responded. Segmental strain analysis of non-scarred segments revealed no difference in peak strain metrics. However, a significant increase in the Time to Peak circumferential strain (ttpEcc) was seen (51.35+/-8.79 versus 45.77+/-7.87% of the cardiac cycle duration) in the response cohort. ttpEcc showed the strongest predictive accuracy for CRT response with an AUC of 0.68 (PPV = 0.79, NPV = 0.60, sensitivity and specificity of 0.79 and 0.60, respectively). Finally, time to peak strain values correlated significantly with percent change in LVESV at 6 months using endocardial strain measures (circumferential: r = -0.35, longitudinal: r = -0.36, minimum principal strain: r = -0.39) and epicardial strain measures (circumferential: r = -0.42, longitudinal: r = -0.33) (p < 0.05 for all).

## Conclusions

Time to peak strain of non-scarred myocardial segments, a measure provided through spatially matched 4D strain and LGE analysis, is predictive of response to CRT and offers a novel approach to assist the selection of patients for this therapy.

